# Microwave Tunable Metamaterial Based on Semiconductor-to-Metal Phase Transition

**DOI:** 10.1038/s41598-017-06230-y

**Published:** 2017-07-18

**Authors:** Guanqiao Zhang, He Ma, Chuwen Lan, Rui Gao, Ji Zhou

**Affiliations:** 1 State Key Laboratory of New Ceramics and Fine Processing, School of Materials Science and Engineering, Tsinghua University, Beijing, 100084 China; 2 College of Applied Sciences, Beijing University of Technology, Beijing, 100124 China; 3 State Key Laboratory of Low-Dimensional Quantum Physics, Department of Physics and Tsinghua-Foxconn Nanotechnology Research Center, Tsinghua University, Beijing, 100084 China; 4 School of Information and Communication Engineering, Laboratory of Network System Architecture and Convergence, Beijing University of Posts and Telecommunications, Beijing, 100876 China; 5 High Temperature Thermochemistry Laboratory, Department of Mining and Materials Engineering, McGill University, Montreal, Quebec H3A 0C5 Canada

## Abstract

A microwave tunable metamaterial utilizing the semiconductor-to-metal transition of vanadium dioxide (VO_2_) is proposed, experimentally demonstrated and theoretically scrutinized. Basic concept of the design involves the combination of temperature-dependent hysteresis in VO_2_ with resonance induced heating, resulting in a nonlinear response to power input. A lithographically prepared gold split-rings resonator (SRR) array deposited with VO_2_ thin film is fabricated. Transmission spectra analysis shows a clear manifestation of nonlinearity, involving power-dependence of resonant frequency as well as transmitted intensity at both elevated and room temperature. Simulation performed with CST Microwave Studio conforms with the findings. The concept may find applications in transmission modulation and frequency tuning devices working under microwave frequency bands.

## Introduction

Owing to the ever-growing advances in the burgeoning field of metamaterials, the world is introduced to a whole spectrum of engineered structures and devices with features so unique that are never observed in nature. With elaborately designed size, shape and repetitive array of the “meta-atoms”, various exotic electromagnetic responses could be achieved. Such properties not only involve the famous examples of negative-index materials^1^, perfect lens^2^ and their use in cloaking devices^3^, but also a rich variety of appliances in the field of telecommunication, a critical part of our social infrastructure, built around devices with tuning and modulation capacities. In the effort to maximize their capabilities, functional materials with intriguing specialities are frequently utilized in the building blocks for metamaterials. Among which, stands out the vanadium dioxide, a material capable of sharply altering its electromagnetic properties through a process known as semiconductor-metal phase transition, which happens at a rather moderate temperature. Such feature endows vanadium oxide with high potentialities in its application as electromagnetic functional materials, along with a number of possible tuning and modulation applications.

VO_2_ behaves like semiconductor under its phase transition temperature Tc = 68 °C with monoclinic crystal structure. When heated above that point, crystal structure changes to rutile, accompanied with a dramatic increment of conductivity by several orders of magnitude, which signifies the occurrence of a metal-like state^4–7^. Such phase transition can be induced thermally^8^, electrically^9^ and optically^10^. For this reason, the combination of VO_2_ thin film or small particles with multifarious metamaterial structures, such as split-ring resonators^11^, are regarded as, and often proven to be, an efficient way towards dynamically controlling electromagnetic properties. Typical focus of attention concentrates on several subfields of research, including tunable or switching metamaterials^12–19^, transmission modulation^20, 21^, perfect absorber/emitter^22–26^, hyperbolic metamaterials^27, 28^, memory metamaterials^29, 30^, etc. However, the vast majority of existing studies are carried out in the frequency range of terahertz or far-infrared, leaving microwave VO_2_ metamaterials a largely uncharted area for exploration. It is a non-negligible fact that metamaterials working under microwave frequency range bear broad research and utilization prospects, and as a matter of fact, power-tunable microwave metamaterials have already been on the rise for drawing the very latest academic attention. Pendry *et al*. had foreseen the possibility of enhancing nonlinearity by introducing nonlinear elements into the gap of split-ring resonator where electromagnetic field reaches its maxima^31^. Wang *et al*. implemented such proposal and measured the power-dependent scattering parameters of varactor-loaded split-ring resonators in microwave frequency bands^32^.

Here we report a power-tunable VO_2_ metamaterial under microwave frequency band, based on the concept of combining VO_2_ semiconductor-metal phase transition with SRR array. VO_2_ thin film is deposited onto gold split-rings by means of magnetron sputtering. Scattering parameters are measured and the results clearly indicate a variation of resonant characteristics in correlation to incident power and the resulting heat-induced phase transition of VO_2_, confirming its nonlinear property. A temperature-dependent hysteresis curve of the metamaterial’s transmission property under microwave frequency bands is also revealed. Numerical simulation further validates the experimental results.

## Results

### Sample designs

**Figure 1:**
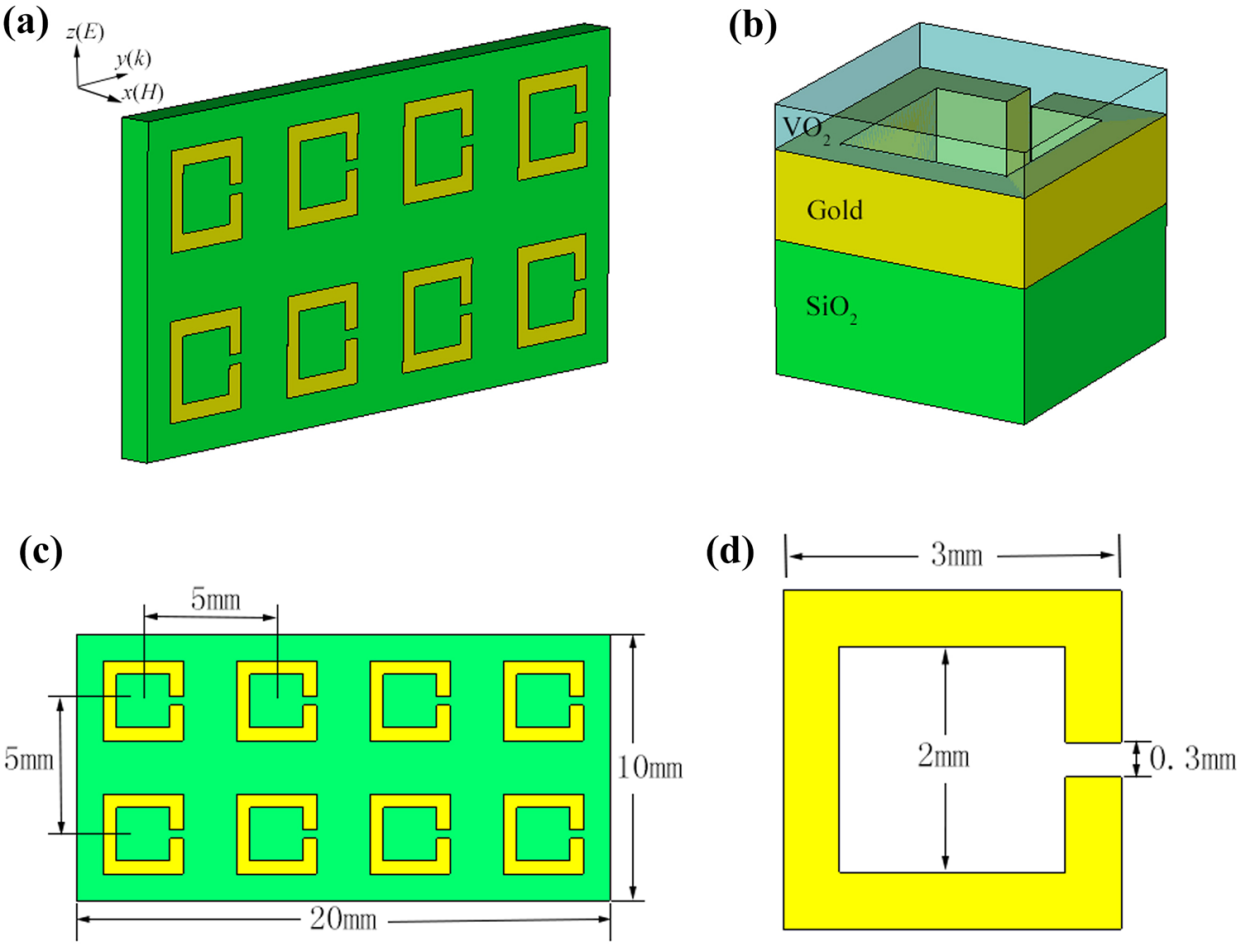
Power-tunable microwave VO_2_ metamaterial designs. (**a,b**) Schematic view of the sample that consists of a SiO_2_ substrate, VO_2_ thin film and gold split-rings. (**c,d**) Size parameters of the sample.

Schematic view of the power-tunable microwave VO_2_ metamaterial studied in this paper is shown in Fig. 1(a) and (b), which consists of a SiO_2_ substrate, gold split-ring resonators, and a thin layer of VO_2_ film. The thickness of SiO_2_ substrate and gold split-rings are 1 mm and 200 nm, respectively. VO_2_ thin film is deposited onto gold split-rings with a thickness of 150 nm. Detailed dimensions of the metamaterial sample are presented in Fig. 1(c) and (d). The size of SiO_2_ substrate is 20 mm × 10 mm, with eight split-rings evenly distributed on its surface. The spacing between adjacent split-rings in y and z direction are both 5 mm. Square split-rings are employed in this study, with outer side length, inner side length and opening gap of 3 mm, 2 mm and 0.3 mm, respectively.

### Experimental setup and measured results

The metamaterial sample is inserted into a rectangular X-band (8~12 GHz) waveguide to obtain scattering parameters. Such waveguide has a cross section of 22.86 mm × 10.16 mm and can support TE_10_ mode microwave wave propagating inside it. X-axis, y-axis, and z-axis are defined as the directions of the orthogonal magnetic field, wave vector and electric field, respectively, as illustrated in Fig. 1(a). The x-axis is perpendicular to the surface of split-rings and y-axis is parallel to the opening gaps. Electromagnetic signal is generated from a vector network analyzer (Agilent N5230C) on one of its two ports (Port 1) and is received on the other (Port 2). By connecting a power amplifier (TH1466C) to Port 1, electromagnetic signal from the vector network analyzer can be amplified and scattering parameters under different incident microwave power levels can thus be easily characterized. An X-band attenuator is also connected to Port 2 to prevent the instrument from potential damage.

**Figure 2:**
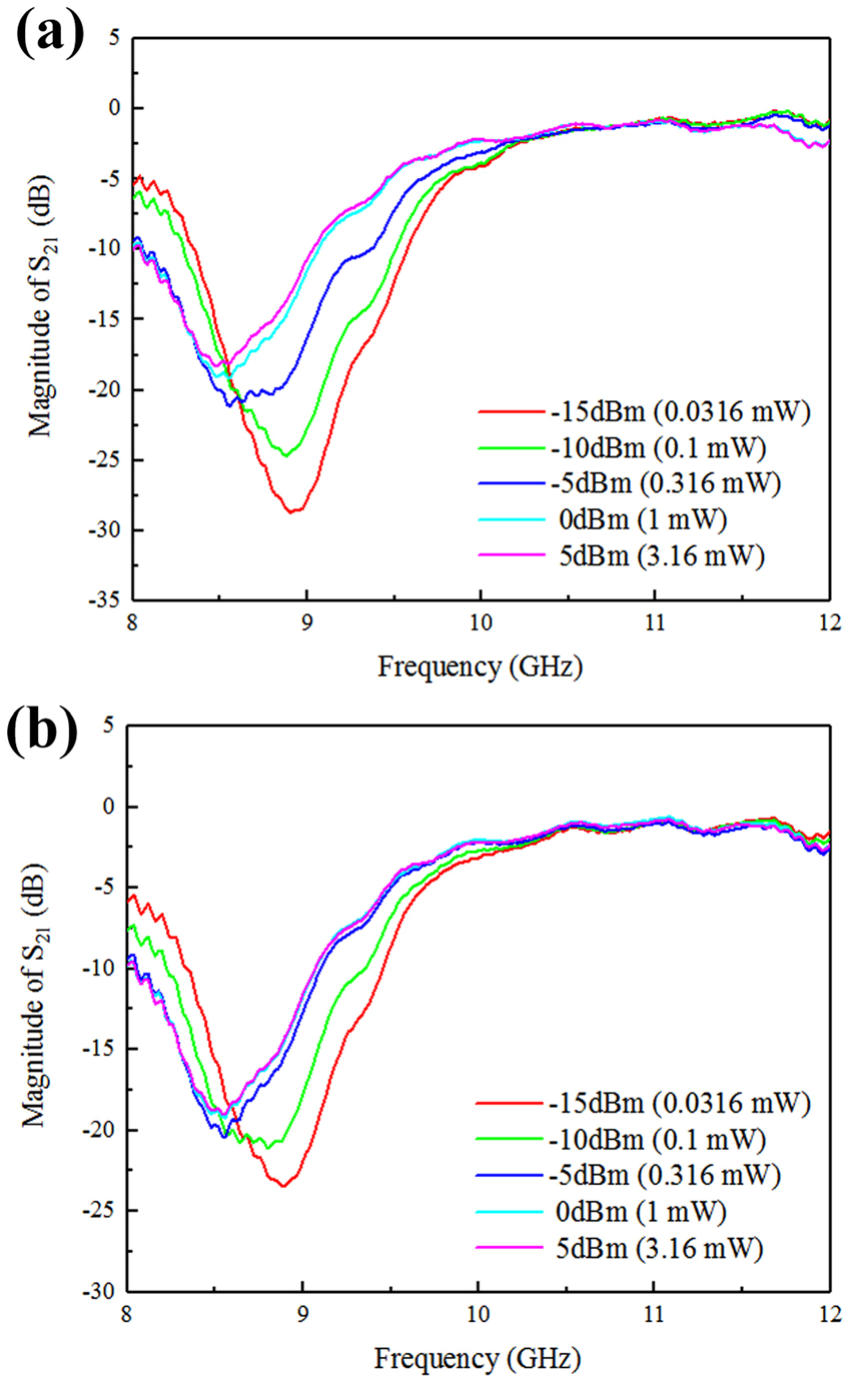
Measured transmission spectra under different incident microwave powers. (**a**) at room temperature (no bias temperature). (**b**) at a bias temperature of 50 °C.

Figure 2(a) shows the transmission spectra of the VO_2_ metamaterial under different incident signal powers (−15 dBm~5 dBm) at room temperature (25 °C). All of the curves are measured after sufficiently long time so that a stable state could be reached. As incident microwave power is increased, the resonant frequency shifts towards lower frequency region and the magnitude of resonant peak decreases sharply. When the incident power is −15 dBm, the lowest power setting for our instruments, the resonant frequency is 8.90 GHz and the magnitude of resonant peak is −28.7 dB. As the incident power is increased to 5 dBm (highest level available), the resonant frequency changes to 8.48 GHz and the magnitude of resonant peak changes to −18.3 dB. Such noticeable variation of transmission property upon different incident signal powers is a clear manifestation of nonlinearity in microwave frequency band, and also a demonstration of its power-tunable behavior and controllability in its transmission modulation performance.

The mechanism of such nonlinear behavior can be attributed to the unique semiconductor-metal phase transition found in VO_2_. When the incident microwave power is set at −15 dBm, which is nearly the minimum signal power of vector network analyzer, the electric field and induced current inside the gold split-rings are relatively small so that the ohmic loss remains at a lower level. The heat generated by split-rings would quickly be dissipated into the surroundings and the temperature of the metamaterial would not rise above the critical temperature. Therefore, the VO_2_ thin film remains in semiconductor state. With the increase of incident microwave power, the ohmic loss multiplies, inducing a heat build-up and elevating the temperature of the metamaterial until thermal equilibrium with the surroundings is reached. When heated above the phase transition temperature, VO_2_ would turn into metallic state and the conductivity sees a dramatic boost, leading to the change of transmission properties of the metamaterial.

The magnitude of S_21_ is defined as the square root of power delivered to port 2 divided by power incident on port 1, and is commonly adopted to characterize power transmittance. In Fig. 2(a), the S_21_ curve shows little variation when increasing the power level from 0 dBm to 5 dBm. This might signify that the temperature of the metamaterial is sufficiently high and the phase transition of VO_2_ is already completed. Therefore, the conductivity of VO_2_ would almost hold constant even if the ambient temperature or incident power is further increased. And the transmission property would remain stable correspondingly. In order to verify such speculation, the transmission spectra of different incident microwave powers under a bias temperature of 50 °C are measured. A heating belt is wound around the surface of rectangular waveguide and a temperature-control system is connected to the heating belt to provide precise control over the heating current and subsequently the system temperature. With such bias temperature, the ohmic loss of split-rings induced by incident electromagnetic power would easily elevate the temperature of the metamaterial above the phase transition point of VO_2_ thin film. The results are shown in Fig. 2(b). When the incident power is −15 dBm and −10 dBm, the magnitude of resonant peaks decrease, compared to Fig. 2(a) where no bias temperature is applied, indicating that the bias temperature contributes to the change of transmission property when incident microwave power is relatively lower. Yet when the incident power is 0 dBm and 5 dBm, the magnitude of resonant peaks remains steady, suggesting that the temperature of the metamaterial is sufficiently high even without bias temperature applied, and the phase transition of VO_2_ can complete thoroughly by incident power alone.

**Figure 3:**
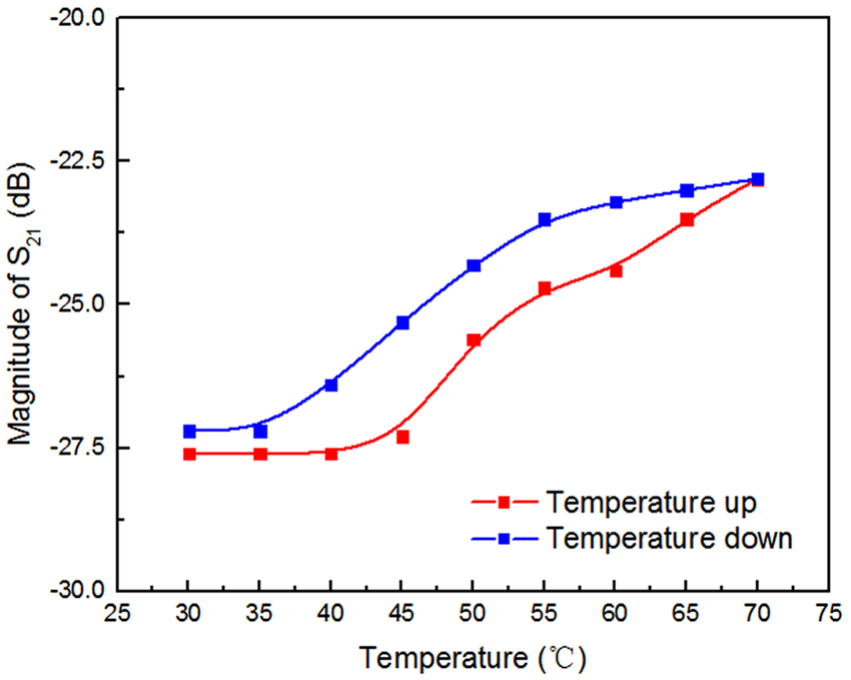
Measured temperature-dependent hysteresis curve of transmission at 8.88 GHz with incident power set to −15 dBm.

The temperature-dependent behavior of VO_2_’s conductivity is a typical hysteresis loop^4^, which means that the conductivity is a double-value function of temperature. Therefore, one may speculate that the property of metamaterial has also a hysteresis relationship with temperature. Figure 3 shows the temperature-dependent hysteresis curve of the metamaterial’s transmission property at 8.88 GHz when the incident power is −15 dBm with red line indicating ‘temperature-up’ and blue line indicating ‘temperature-down’. Data is collected with a step of 5 °C. The absolute value of S_21_ magnitude is larger when increasing temperature than that when decreasing temperature, which is a clear manifestation of hysteresis behavior. The critical temperature of the metamaterial in Fig. 3 is around 50 °C, lower than the VO_2_ phase transition temperature, which can be explained as follow. The ‘temperature’ defined on the horizontal axis of Fig. 3 is actually the temperature measured on the outer surface of rectangular waveguide as the temperature inside the waveguide cannot be easily obtained. Due to the heat exchange with environment, the temperature on the outer surface of waveguide is lower than that inside the waveguide where most of the heat is trapped. Thus the phase change has already taken place when the temperature of the outer surface reached 50 °C.

### Numerical Simulation Results

**Figure 4:**
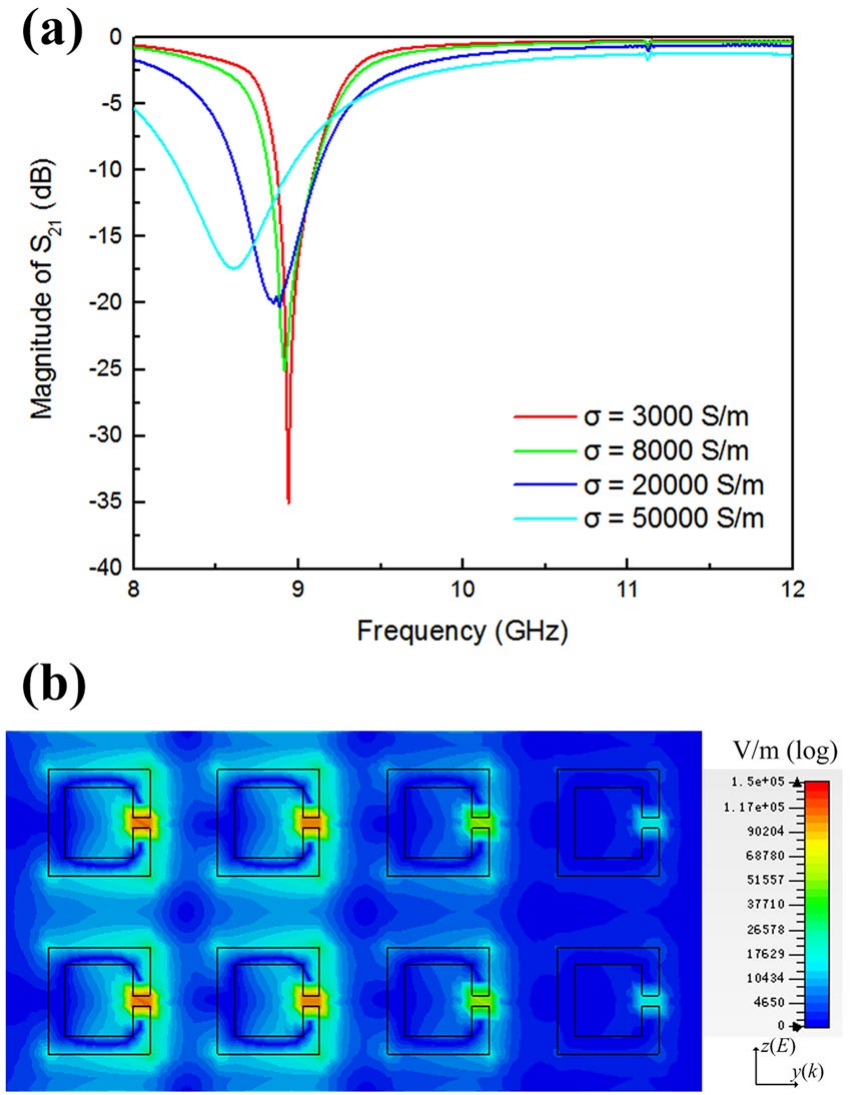
Numerical simulation results. (**a**) Transmission spectra when altering the conductivity of VO_2_ (σ). (**b**) Electric field distribution at σ = 3000 S/m.

To further corroborate experimental results, electromagnetic simulation is carried out using commercial software, the CST Microwave Studio, as an auxiliary explanation. All parameters are set to be consistent with those in the experiment. Figure 4(a) shows the transmission spectra of the metamaterial for VO_2_ conductivity ranging from 3000 to 50000 S/m. The resonant frequency undergoes a red-shift and the magnitude of resonant peak decreases as the conductivity increases, which coincides with what was previously observed in experiment. Figure 4(b) shows the electric field distribution when the conductivity of VO_2_ is set as 3000 S/m. The electric field around the opening gap sees a colossal buildup and the ohmic loss would facilitate the elevation of temperature, resulting in a heat-induced semiconductor-metal phase transition in the VO_2_ film. Field intensity near the right side of the metamaterial is much lower than that on the left side, since the electromagnetic signal originates from the left side, as the coordinate axes indicate. Electromagnetic wave energy would attenuate along the direction of the wave vector.

## Discussion

The VO_2_ metamaterial demonstrates clear power-tunable property in the X-band region. Due to the intrinsic electromagnetic property of split-ring resonator, intense electric field is generated around the opening gap, in response to incident power. When this is coupled with VO_2_, the resulting heat-induced semiconductor-metal phase transition enables a microwave-tunable response, represented by shifts in resonant frequency, along with variations in the magnitude of resonant peak, upon altering the incident electromagnetic signal power. The behavior is validated both through experimental measurements of transmission spectra and numerical simulations.

## Conclusions

In this study, we have proposed a power-tunable metamaterial based on the heat-induced semiconductor-metal phase transition in VO_2_. The concept is realized in conjunction with split-ring resonators, and a microwave-tunable response involving shifts in both resonant frequency and resonant peak intensities have been observed. The findings are further confirmed by numerical simulations. Such power-tunable nonlinear behavior might be of potential applications for frequency tuning and transmission modulation devices working under microwave frequency bands, given the rich flexibilities with metamaterial designs based on such concept. Moreover, the temperature-dependent hysteresis curve of S_21_ value is a representation of the VO_2_ conductivity’s temperature-dependent hysteresis performance, which may find applications in memory devices featuring microwave frequencies.

## Methods

### Sample fabrication

The split-ring resonators are fabricated by standard lithography, high-vacuum deposition of 200 nm gold onto a SiO_2_ substrate and subsequent lift-off. VO_2_ coating with a thickness of 200 nm is then deposited onto split-rings via DC magnetron sputtering technique from a pure vanadium target and a carefully controlled Ar/O_2_ gas mixture. The percentage of O_2_ is set to be 2% in the reactive environment of Ar/O_2_ gas mixture with total pressure in the chamber around 0.6 Pa. The sample is later annealed in O_2_ atmosphere at 450 °C for 10 minutes to obtain desired phase change property.

## Data Availability

All data generated or analysed during this study are included in this published article.
